# Third Annual Meeting of the Alumni Association of the Medical Department of the University of Buffalo

**Published:** 1877-03

**Authors:** 


### Third Annual Meeting of the Alumni Association of the Medical Department of the University of Buffalo.

The Third Annual Meeting of the Alumni Association of the Medical Department of the University of Buffalo, was held in this city February 20, and in all respects was as important as any of its predecessors. In the evening the Annual Commencement of the Medical College took place at St. James Hall. A supper followed in the reading room of the Young Men's Association, and a day's good work was succeeded by a season of festivity which the guests will remember long and pleasantly.

The addresss before the Alumni Association, by Prof. Frank H. Hamilton, of New York City, is of so much interest and is so suggestive to the profession generally that we must indulge in publication of some of the paragraphs even though the newspapers have had the first publication. After a beautiful introduction he says:

Gentlemen, my visit here is especially to meet and talk with you; but so many thoughts obtrude themselves after my long absence from the city that it seems impossible for me to give to you that undivided attention which, as your guest, you have a right to claim. Moreover, I feel myself at a loss what, in the brief time allotted to me, it is best to say. Most that I have learned in the interval of time which has separated us you have learned also. Our science has made progress, but you have kept pace with it. I have had personal experience and so have you. I can no longer take you into the old ampitheater and swing around the circle having all the talk and opinions on my own side—enforcing those statements which are difficult to understand with "demonstrstions" equally difficult to see. You are here as my peers, and I feel diffident of my ability to either instruct or entertain you. Please indulge me then in my caprices, and permit me to say whatever comes uppermost.

### SPECIALTIES IN MEDICINE AND SURGERY.

The question of specialties in medicine and surgery has of late attracted a good deal of attention and given rise to considerable discussion. The question is, are they useful; do they tend to advance our science? I answer yes and no. There are two sides to this question as there are most other questions. Within certain limitations and upon certain conditions they are useful, and the converse is equally true. To understand this matter, let us see what has happened within a few years. When I commenced the practice of my profession, and within your recollection, we, who simply called ourselves Physicians and Surgeons—general practitioners—occupied the field. There might have been here and there a strolling specialist, but they failed generally to secure the confidence of the people or to make any dangerous inroads upon our practice. Our legitimate right to the entire territory was practically undisturbed. Since then, however, following upon a few established successes, there has sprung up suddenly a great excitement, such as inevitably follows the rumors of the discovery of a new bonanza; and emigrants, with no other capital than a pick and gun, have crowded into the territory, and never so much as saying "by your leave," they have selected their ground and driven their stakes until little or nothing is left to the original proprietors, but the barren title of Doctors, and which title is found to have no weight in law as against the intruders. To-day there is scarcely an organ of the body or an anatomical or classified structure into which their stakes have not been driven, and from which trespassers are not warned. Let us mention

### A FEW OF THESE SPECIALTIES.

For the head there is the occulist; the aurist; the catarrh doctor for the nose; the throat doctor; the dentist, of whom there are several subordinate and separate departments.

There are heart doctors, and lung doctors, and some who include both of these important organs in their practice, and might properly be called chest doctors. The liver is an old claim, which was worked a good many years and then abandoned as being worked out. There are kidney doctors, also; but no one has ever driven a stake into the spleen, although I do not see any good reason why it might not be worked profitably. There are a great many people who are spleeny and if any one could summon the courage to put up a sign, I am sure he would be well patronized.

There are doctors for the nerves and for the blood; doctors who can expel by their medicines all the bad humors and leave their patients in good humor. There are skin doctors and bone doctors, spine doctors and crooked-leg doctors, natural bone-setters, hair doctors and corn doctors.

There are doctors for women, also, as distinguished from doctors for men: but there are no doctors for men as distinguished from women. We do not feel offended at this act of partiality toward women. Bless their souls, no. So far as we are concerned, we would be quite willing they should have all of the doctors, and that we should be left to suffer and die without their help. But one cannot help asking whether, if it be true that the *cordæ tendinæ* of women's hearts are more delicate than ours, and their nerves are attuned to a higher key—whether admitting this and much more, there is really any more difference between a woman and a man than between a man and a woman; or whether the establishment of the specialty of a woman doctor, means that a woman is a man *plus* a woman, and that a general practitioner cannot be expected to attain to a knowledge of the *plus?* I have only this morning seen an advertisement in a Syracuse paper, in which a Homœpathic doctor announces that he makes a specialty of children. Very small ones I suppose.

I have not, by any means exhausted the list of specialties which are to-day recognized in medicine and surgery; but I think you will see that there is a good deal of crowding, packing edgewise and some overlapping, and that not much chance is left for the old-fashioned physician and surgeon.

The condition of matters will be best illustrated by a supposed case; and which I shall take care not to exaggerate very much beyond what has repeatedly come under my own observation.

A lady called upon a physician for advice, stating that her health was "completely broken," and that she was "affected with a frightful collection of maladies," but as she did not specify them particularly, the doctor began to interrogate her about the condition of her nervous system; when she replied promptly that he need not trouble himself to inquire into that matter, as she had consulted Dr. Brown Sequard, and under advice she had taken strychnine and iron, and phosphorous to build up her nerves. The doctor then suggested that he would like to examine her heart. "Oh," said she, "that is in a very bad condition, but Dr. Clark is giving me aconite, belladonna and digitalls to lower its action." "Your lungs," said the doctor inquiringly. "Dr. Metcalf has examined my lungs," was the ready reply, "and I do not care for any other advice in that matter. He is giving me several cough mixtures." "But I notice," said the doctor, hoping that he had hit upon the weak point, and the one which she proposed to entrust to his special guardianship, "I observe that you have still some cough, and perhaps the trouble is in your throat rather than your lungs." "Dr. Metcalf thought this might be so," said she, "and he sent me to Dr. Elsburg, who says there is trouble there, also, and he is applying daily iodiform spray, and other things." "You wear glasses?" "Yes, my sight was a little affected, but Dr. Noyes thinks they will be cured if I wear glasses and take his medicines long enough."

"Pray, madam," exclaimed the Doctor completely baffled in his attempt to find an unprotected point in this splendid line of defenses, "will you kindly inform me what organ, or fragment of an organ, or tissue you wish to place under my charge, or consult me about?" "Yes, Doctor, if you please. I have consulted a good many other specialists, and as I am abundantly able to do so, and as health is of more value than money, I propose to consult them all. I know you by reputation very well and I felt sure you could tell me better than any one else who makes a specialty of warts, and I would thank you to give me a letter of introduction to whoever you think is best. I have a wart on the back of my neck, and as I would not like it to leave a scar, you will please recommend me to some one who will cure it without cutting."

